# Carbon Dot-Based Sensors for Food Chemical Hazard Detection: Real-Food Applications, Sample Preparation, and Portable Analysis

**DOI:** 10.3390/foods15193548

**Published:** 2026-10-05

**Authors:** Xueling Cao, Wenwen Pang, Guohui Li, Danfeng He

**Affiliations:** College of Science, Qiongtai Normal University, Haikou 571100, China

**Keywords:** carbon dots, carbon quantum dots, food chemical hazards, real food matrices, sample preparation, portable detection, smartphone

## Abstract

Carbon dots (CDs) are a heterogeneous family of fluorescent carbon nanomaterials that includes carbon quantum dots (CQDs) as a subclass. Owing to their favorable fluorescence, surface chemistry, and functionalization potential, CDs have been widely investigated for sensing analytes relevant to food chemical safety. This structured narrative review and evidence map examines a broad analytical corpus of 106 original CD-based studies published between 2016 and 29 August 2026, covering heavy metals and metalloids, pesticides, veterinary drugs and antibiotics, and food additives, adulterants, and processing contaminants. Food-matrix eligibility was then assessed separately: 68 studies experimentally applied the method to an actual food matrix and generated 98 study–food matrix combinations. This two-tier analysis shows that veterinary-drug studies focused mainly on dairy products, pesticide studies predominantly addressed fruits and vegetables, and the comparatively limited food applications for heavy metals concentrated in aquatic products. Centrifugation, filtration, organic-solvent extraction, and deproteinization remained common, indicating that rapid sensor response does not necessarily translate into rapid end-to-end food analysis. Paper-based, hydrogel, and smartphone-assisted platforms are advancing CD detection from visual observation toward calibration-based quantification; however, validation using commercial samples at native or unknown concentrations, incurred samples, cross-device consistency, and field standardization remain insufficient. The synthesis therefore identifies analytical reliability, complex-matrix compatibility, simplified pretreatment, multi-analyte detection, and standardized on-site quantification as priorities beyond the pursuit of lower detection limits.

## 1. Introduction

Chemical hazards in food mainly include heavy-metal and metalloid contaminants, pesticide residues, veterinary-drug and antibiotic residues, food additives, adulterants, and processing contaminants [1,2]. These substances may enter food systems during cultivation, animal production, processing, storage, and transportation. Therefore, sensitive, rapid detection methods applicable to complex food matrices are essential for food-safety monitoring.

Chromatographic, mass-spectrometric, and atomic-spectrometric techniques offer high accuracy and selectivity, but they generally require specialized instrumentation, laboratory facilities, and relatively complex sample preparation, thereby limiting their use in rapid on-site screening and high-frequency monitoring. Carbon dots (CDs)—an umbrella term for structurally and optically heterogeneous carbon-based nanodots—exhibit good water dispersibility, tunable fluorescence, abundant surface functional groups, and high functionalization potential [3]. Carbon quantum dots (CQDs) constitute one subclass of CDs and should not be used as a synonym for the entire family. CD materials have been widely investigated for detecting food chemical hazards [4] and have increasingly been combined with ratiometric fluorescence, dual-mode detection, paper-based supports, and smartphone image analysis.

Existing studies show that CD sensing has progressed from proof-of-concept experiments in standard solutions to applications in real food matrices, including milk, eggs, meat, fruits and vegetables, aquatic products, grains, and processed foods. HPLC, LC-MS/MS, GC-MS, ICP-MS, and standard colorimetric methods have also begun to be used for comparative validation. Meanwhile, some platforms have enabled portable detection on paper, hydrogels, or other solid supports [5] and have established calibration relationships using RGB values, color-channel ratios, or other digital signals [6], thereby enabling quantitative readout rather than relying solely on visual observation.

Nevertheless, a clear gap remains between CD-based food detection and routine practical application. Although many systems exhibit low limits of detection in standard solutions, analysis in real food matrices often still requires homogenization, centrifugation, filtration, deproteinization, organic-solvent extraction, or even acid digestion. Food validation also remains dominated by spiked real-food samples, whereas commercial samples at native or unknown concentrations, naturally contaminated or incurred samples, and certified reference materials are comparatively scarce [7]. Although paper-based and smartphone platforms improve portability, carrier-to-carrier variation, illumination conditions, camera settings, and image-processing procedures may still affect quantitative results [8]. Previous reviews have focused mainly on CD synthesis, optical properties, and sensing mechanisms, whereas the real-food applicability, sample-preparation burden, validation strength, and portable quantification of CD-based methods across different food chemical hazards have not been systematically compared.

Accordingly, this review addresses three linked questions: (i) which food-relevant chemical hazards have been detected using CD-based sensors; (ii) how convincingly their analytical performance has been demonstrated in actual food matrices, considering sample preparation and validation quality; and (iii) how far these systems have progressed from laboratory proof-of-concept assays toward calibration-based portable quantification. The 106-study broad analytical corpus is used to characterize publication and sensor-design trends, whereas claims about food applicability are based on the 68-study food-matrix subset. Literature retrieval and evidence organization were informed by PRISMA 2020, PRISMA-S, and predefined evidence-mapping categories. The review then compares hazard–matrix patterns, sample-preparation burden, validation approaches, and portable-platform maturity to identify both demonstrated advances and remaining translational gaps.

## 2. Literature Search and Data Organization

### 2.1. Literature Search and Review Scope

This study adopted a structured evidence-mapping review design. Iterative searches were conducted through PubMed/PubMed Central, DOI/Crossref metadata, and publisher platforms, supplemented by reference tracking, citation tracking, and targeted full-text retrieval. Search terms combined carbon dots, carbon quantum dots, CDs, and CQDs with terms related to the four target-hazard categories. Candidate records underwent deduplication, title and abstract screening, full-text retrieval, document-identity verification, and eligibility reassessment. Ultimately, 106 original studies with complete full text were included in the broad analytical corpus, together with 14 reviews and methodological or portable-translation studies used for background and discussion. Literature screening and process reporting drew on the transparent-reporting principles of PRISMA 2020 [9], while search documentation was informed by PRISMA-S [10].

### 2.2. Inclusion and Exclusion Criteria and Study Classification

Studies were eligible for the broad analytical corpus if they met all of the following criteria: (1) original experimental research; (2) a carbon-dot material served as a functional component for recognition, signal generation, or signal transduction; (3) the target belonged to one of four categories—heavy metals and metalloids, pesticides, veterinary drugs and antibiotics, or food additives, adulterants, and processing contaminants; (4) analyte detection was the main objective, rather than only material synthesis, adsorption, removal, or degradation; and (5) the full text contained sufficient information to extract sensing and application data. Duplicate reports, conference abstracts with insufficient information, review articles, and studies outside the predefined hazard scope were excluded. In this review, CD is used as the umbrella term, while CQD denotes a subclass rather than a synonym for all CDs. Quantum-confinement behavior was not inferred when an original study did not provide supporting evidence; therefore, cross-study synthesis and table terminology were normalized to CD, while source titles remain unchanged in the reference list.

Studies were assigned exclusively to their primary food-safety target: heavy metals and metalloids (23 studies), pesticides (27 studies), veterinary drugs and antibiotics (29 studies), or food additives, adulterants, and processing contaminants (27 studies). Studies targeting multiple analytes within the same broad hazard class were counted once in that class, while compounds tested only for selectivity or interference were not treated as additional targets. No included study provided equivalent food-matrix validation for co-primary analytes spanning different hazard classes.

### 2.3. Data Extraction, Coding, and Statistical Analysis

The following information was extracted from the full texts: publication year, hazard category and target analyte, CD sensing system, detection/readout mode, linear range, limit of detection (LOD), food matrix, sample preparation, recovery, relative standard deviation (RSD), reference method, portable support, smartphone or portable device, and quantification method [11,12]. Real-food evidence was further coded as spiked food samples, commercial samples analyzed at native or unknown concentrations, experimentally treated or incurred samples, and certified reference materials. The main limitations of representative studies were summarized according to sample-processing burden, matrix complexity, real-sample type, and independent reference-method validation.

The evidence base was organized into two tiers. The broad analytical corpus comprised 106 original CD-based sensing studies targeting analytes relevant to food chemical safety, irrespective of whether validation was conducted in standard solutions, water, non-food matrices, or foods; this corpus was used only to describe publication and sensor-design trends. The food-matrix subset comprised 68 studies in which the method was experimentally applied to an actual food matrix. Only this subset contributed to the real-food synthesis, food-matrix distribution, sample-preparation statistics, and conclusions concerning food applicability. Studies tested only in standard solutions, water, or other non-food matrices—including representative examples in Refs. [13,14]—were classified as contextual evidence and were not treated as food applications.

**Figure 1:**
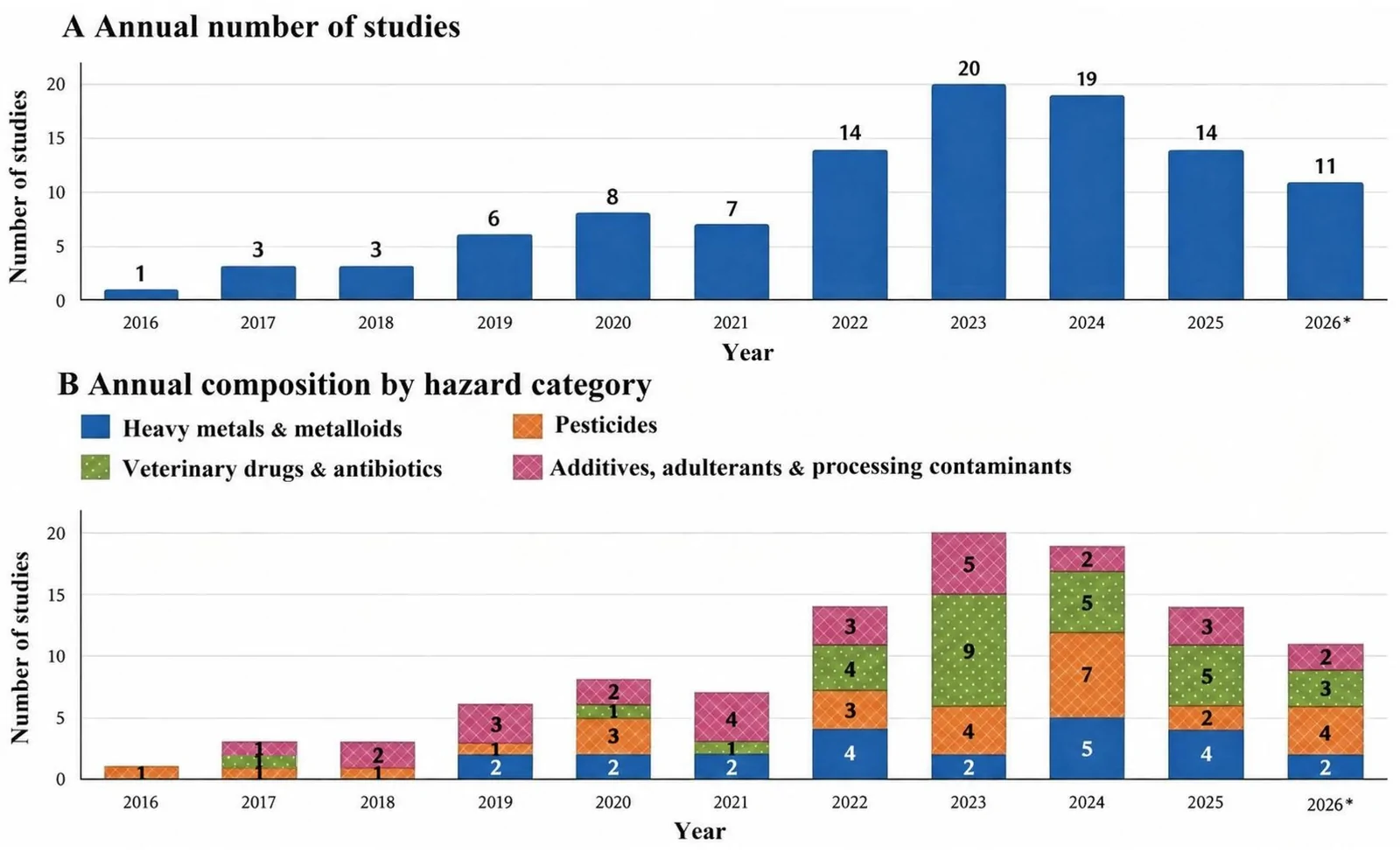
Temporal trends in the broad analytical corpus of CD-based sensing of food-safety-relevant analytes. (**A**) Annual number of studies. (**B**) Annual composition by hazard category. Category counts do not imply food-matrix validation. * Note: Data for 2026 are incomplete and should not be interpreted as a full-year decline.

**Figure 2:**
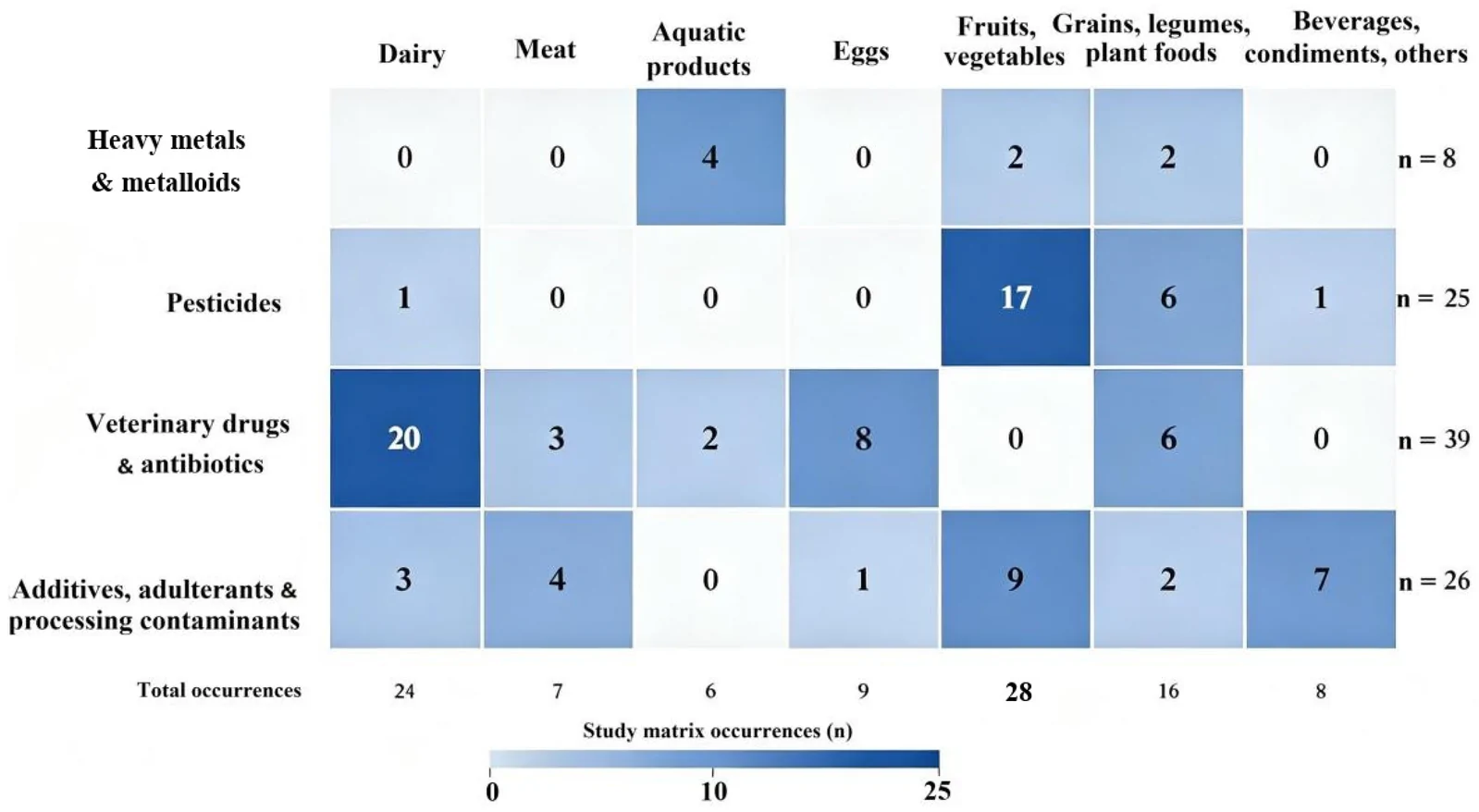
Distribution of CD sensing studies across hazard classes and food matrices. The 68 food-matrix studies generated 98 study–food matrix combinations because one study could include multiple food-matrix categories. Counts represent study–food matrix category occurrences rather than unique study numbers.

**Figure 3:**
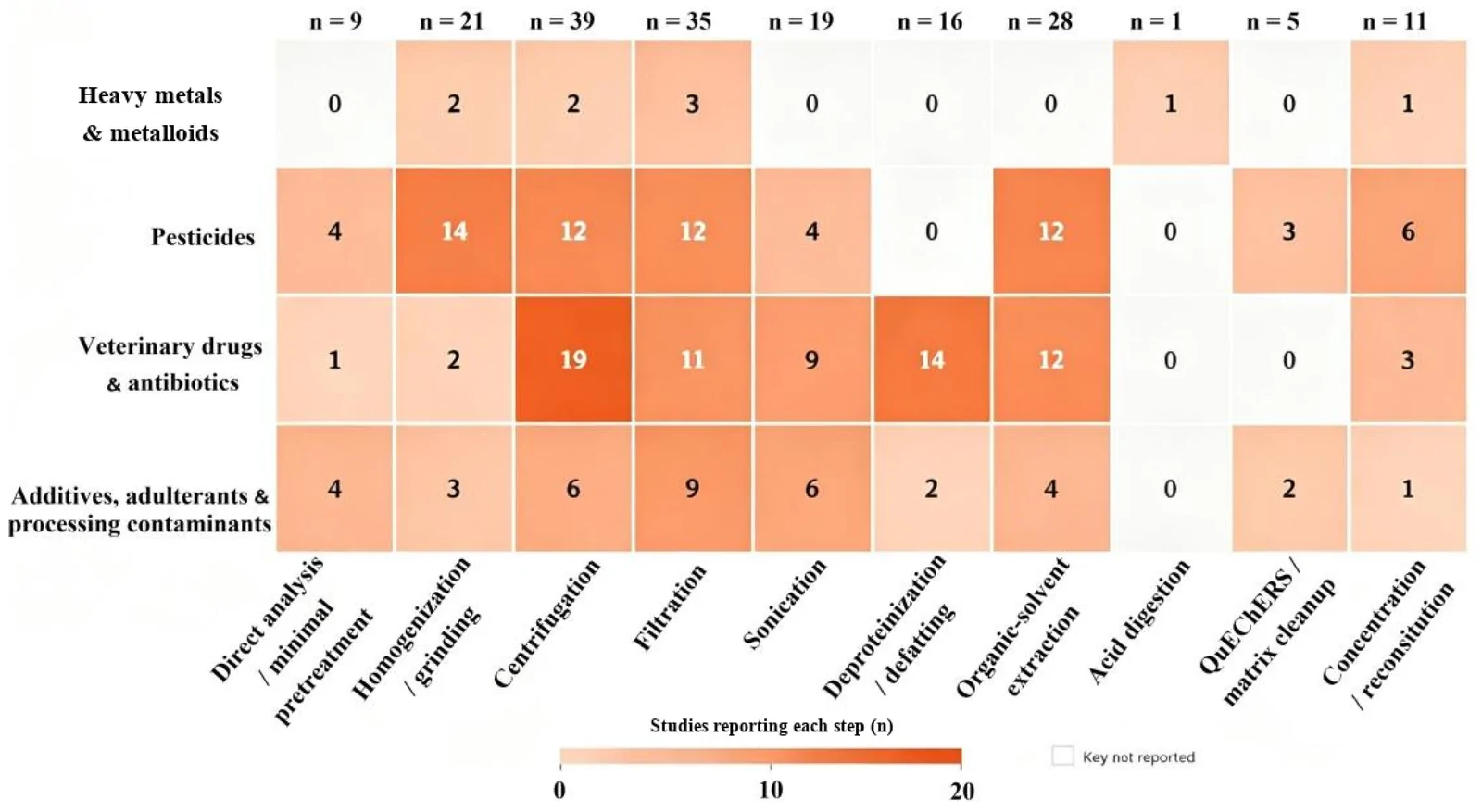
Reported sample-preparation steps in CD-based real-food studies. The 68 food-matrix studies were grouped by hazard class; multiple steps could be reported in one study, so counts across columns are not mutually exclusive. Only explicitly reported steps were counted.

**Figure 4:**
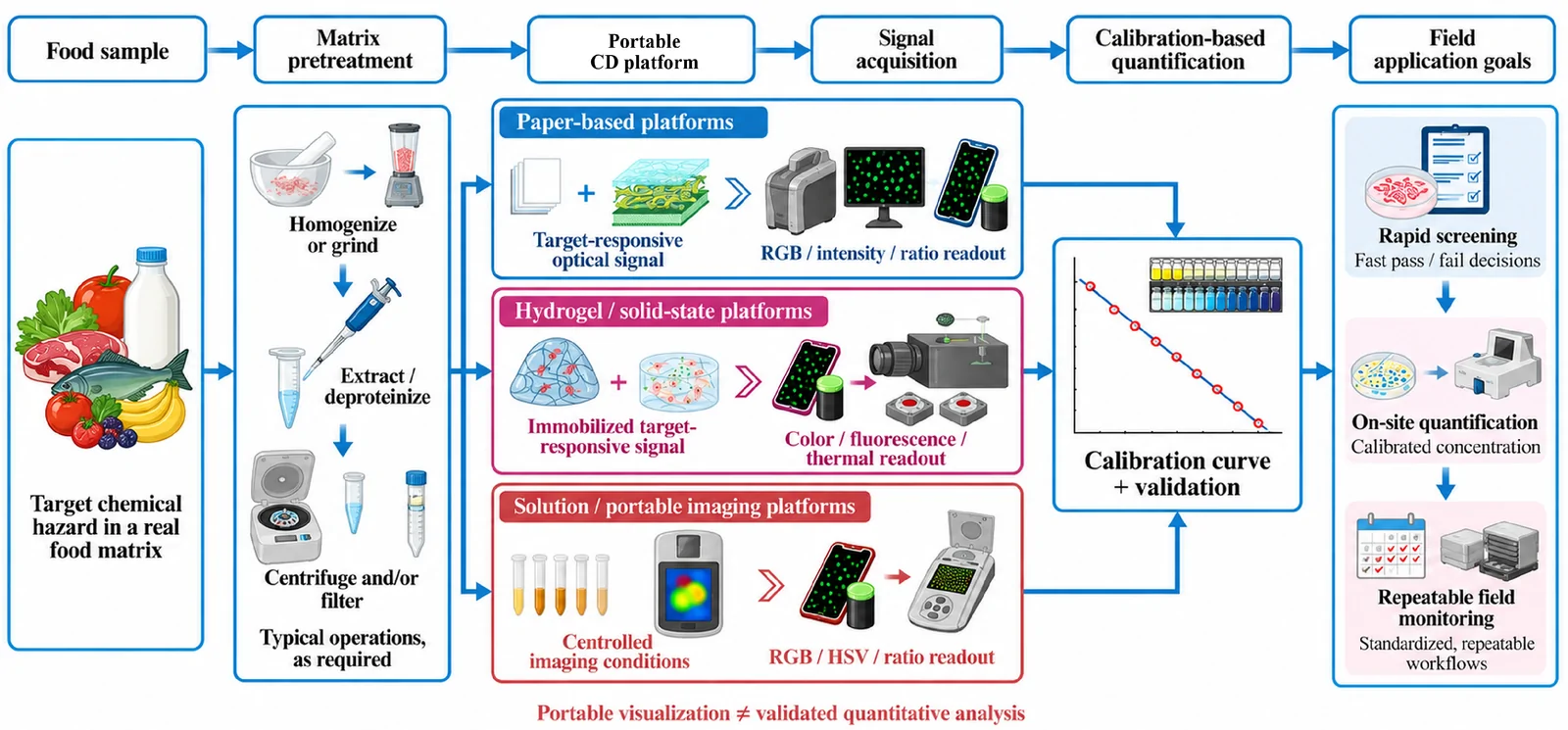
Conceptual pathway for portable CD-based food chemical hazard detection, from matrix pretreatment to calibration-based field analysis.

Figure 1 summarizes annual trends and hazard composition for the broad analytical corpus of 106 original studies; its category counts do not imply food-matrix validation. Data for 2026 were collected only through 29 August and were treated as an incomplete year. Figure 2 is based on the 68 food-matrix studies. When a study involved more than one broad food category, each category was counted as a separate study–food matrix combination, whereas multiple foods within the same broad category were counted only once, yielding 98 combinations. Figure 3 is likewise based on the 68 studies and includes only sample-preparation steps explicitly reported in the original articles. Because a study could include multiple steps, the categories are not mutually exclusive, and missing information was not interpreted as the absence of pretreatment. Figure 4 presents a conceptual pathway from sample processing to on-site quantification for portable CD-based food detection and does not imply that all studies followed the same linear process. Table 1, Table 2 and Table 3 are restricted to representative food-matrix studies and summarize sensing systems, real-food applications, and platforms with calibration-based portable readouts, respectively.

## 3. Carbon-Dot-Based Sensing of Food-Relevant Chemical Hazards

As shown in Figure 1, the 106-study broad analytical corpus grew overall from 2016 to 2023 [1,2], while its scope expanded from scattered early targets to four categories: heavy metals, pesticides, veterinary drugs and antibiotics, and additives, adulterants, and processing contaminants [4]. These annual counts describe the literature corpus rather than the number of studies validated in food. Because the 2026 data represent an incomplete year, they should not be interpreted as evidence of a full-year decline in research output.

Within the reviewed literature corpus, the increase in publications from 2016 to 2023 coincided with growing interest in CD functionalization, food-safety sensing, ratiometric readouts, and portable analytical platforms. The lower counts in 2024 and 2025 may reflect normal annual variation and the specific inclusion criteria of the present review, rather than a sustained decline in the broader field. Because the 2026 data were collected only through 29 August, they were not used to infer a full-year trend.

### 3.1. Common Features and Material Trends of the Reviewed Carbon Dots

Across the 106-study broad analytical corpus, the most commonly reported CD features were aqueous dispersibility, fluorescence emission, abundant surface functional groups, and compatibility with further functionalization or immobilization [3,4]. Hydrothermal or solvothermal synthesis from citric-acid-derived molecules, biomass, and food or agricultural waste was frequently reported. Oxygen- and nitrogen-containing groups, including hydroxyl, carboxyl, and amino groups, were commonly invoked to support analyte interaction, bioconjugation, and dispersion. Nitrogen-doped and multi-heteroatom-doped systems, including N/S- and N/P-doped CDs, recurred across hazard classes, while metal-doped and composite systems were used to generate ratiometric or dual-mode responses.

The cross-study synthesis indicates that the principal commonality lies in fluorescence, surface chemistry, aqueous compatibility, and ease of functionalization—not in uniformly demonstrated quantum behavior. Method development progressed from single-emission solution assays toward dual-emission or ratiometric probes, hybrid nanocomposites, molecularly imprinted systems, immobilization on paper, hydrogel, polymer, or textile supports, and smartphone-assisted readout [5,6]. Because the original literature used carbon dot and carbon quantum dot inconsistently and often did not establish quantum confinement, the present review does not reinterpret all 106 materials as CQDs. Generic cross-study conclusions therefore use the umbrella term CD, which prevents a chemically and structurally heterogeneous literature corpus from being presented as a single quantum-mechanically uniform material class.

### 3.2. Heavy-Metal and Metalloid Contaminants

Heavy-metal and metalloid contaminants are important targets for CD-based food sensing. In the literature included in this review, the principal analytes were Hg^2+^, Pb^2+^, Cd^2+^, Cr(VI), and Ag^+^ [13,14,15,16]. Interactions between metal ions and oxygen-, nitrogen-, or sulfur-containing functional groups on CD surfaces can produce fluorescence quenching, enhancement, or ratiometric changes, thereby providing the signal basis for food analysis.

Within the present literature corpus, food analysis of metal ions covered laver, rice, fish, shrimp, crab, and selected fruits and vegetables. Li et al. applied a CD system to Pb^2+^ determination in commercial laver and validated the same samples by ICP-MS [17]. Shan et al. extended Hg^2+^ analysis to grass shrimp, Chinese mitten crab, grass carp, rice, and other foods [18]. Xu et al. further developed an Ag^+^ platform integrating a paper-based sensor, portable dark box, and smartphone readout, achieving calibration-based quantification in carp and river shrimp [19]. Other work extended metal detection to plant-derived foods such as carrots and potatoes [20].

Within the broad corpus, CD-based heavy-metal sensing ranged from water and model systems—including reports retained only as contextual sensor evidence [13,14,21,22,23]—to actual food matrices [17,18,19,20]. Only the latter informed the real-food synthesis. Compared with pesticide and veterinary-drug studies, food applications remained relatively limited and were concentrated on a small number of metal ions and aquatic foods.

### 3.3. Pesticide Residues

Pesticide residues represent an active area of CD-based detection of food chemical hazards. Targets have included organophosphorus pesticides, glyphosate, acetamiprid, dichlorvos, carbendazim, and several herbicides [24,25,26,27]. Because pesticides vary widely in molecular structure, existing systems frequently use aptamers, enzyme inhibition, ratiometric signals, or paper-based configurations to improve selectivity and detection stability.

Compared with heavy-metal sensing, CD-based pesticide sensing has been applied more frequently to real plant-derived foods. Li et al. used a CD sensing system to monitor pesticide residues dynamically in apples after experimental spraying and compared the results with HPLC [28]. Peng et al. further applied glyphosate detection to time-resolved residue analysis in the leaves, stems, and roots of Chinese chives and confirmed the results by HPLC-MS [29]. Jiao et al. first measured the unknown native level of acetamiprid in commercial lettuce and then performed spike-recovery experiments and LC-MS/MS comparison [30]. Li et al. also developed a CD-labeled aptamer-Ti_3_C_2_T_x_ MXene fluorescence system and validated dimethoate detection in spiked apple juice prepared from commercially purchased fresh apples [31].

Overall, within the literature included in this review, CD-based pesticide detection has been applied relatively broadly to fruits, vegetables, grains, and other plant-derived foods and is beginning to extend toward paper-based supports, portable imaging, and smartphone-based calibration and quantification [32,33]. Han et al. quantified carbendazim using a B/R signal in a fixed dark box with multiple smartphones and compared reading consistency among iPhone, Xiaomi, and Huawei devices [34].

### 3.4. Veterinary-Drug and Antibiotic Residues

Veterinary drugs and antibiotics are among the most extensively studied categories in the present literature corpus, principally including tetracyclines, quinolones, sulfonamides, chloramphenicols, and aminoglycosides. Research was particularly concentrated on tetracyclines [35,36,37,38], with milk, eggs, chicken, and honey representing the most common food matrices.

Existing research has progressed from detecting single drugs to recognizing structurally related antibiotics and applying tiered validation. Lin et al. developed a CD system capable of discriminating several tetracyclines and further evaluated it using a certified reference material for tetracyclines in chicken [39]. Fan et al. achieved concurrent responses to oxytetracycline (OTC), tetracycline (TC), chlortetracycline (CTC), and doxycycline (DOX) and conducted recovery validation in milk and honey [40]; related systems have likewise covered milk and honey matrices [41]. Hu et al. transferred tetracycline detection to filter-paper strips and validated the method in milk, eggs, and chicken [42].

Beyond tetracyclines, sulfadiazine, norfloxacin, malachite green, chloramphenicol, and aminoglycosides have also been investigated in foods such as honey, milk, eggs, meat, and fish [43,44,45,46]. Overall, the veterinary-drug studies included in this review provide a substantial base of real-food applications, although their matrix distribution remains strongly biased toward dairy products.

### 3.5. Food Additives, Adulterants, and Processing Contaminants

Food additives, adulterants, and processing contaminants constitute the most diverse target category in CD-based food detection. Analytes include nitrite, melamine, synthetic colorants, acrylamide, bisphenol A, and curcumin [47,48,49,50]. Because these substances differ substantially in structure and physicochemical properties, the corresponding CD systems also exhibit considerable diversity in detection strategies.

Among representative studies, Zhuang et al. used an N-CD/Fe^3+^ fluorescence-recovery system to detect melamine in spiked raw milk, reporting recoveries of 92.5–105.4% without an independent reference-method comparison of the same samples [51]. Acrylamide detection was subsequently extended to processed foods such as bread [52]. Feng et al. expanded nitrite detection to an Au@CDs-MXene electrochemical sensing configuration [53], whereas Wei et al. integrated aptamer detection, QuEChERS extraction, and LC-MS/MS validation for acrylamide analysis in bread [54].

CD systems have also been used to detect colorants or additives such as Allura Red, Tartrazine, New Coccine, Sudan I, and curcumin in beverages, confectionery, chili powder, tomato sauce, and spices [55,56,57,58]. Overall, this category covers the most diverse food matrices and has progressed from single spiking experiments to the analysis of selected commercial samples at native or unknown concentrations. The analytical performance of representative CD sensors with food-matrix validation is summarized in Table 1.

Taken together, the four hazard categories show different forms of analytical maturity rather than a single uniform progression. Heavy-metal sensing contains substantial contextual evidence from water and model systems, veterinary-drug sensing contributes the densest food-matrix evidence, pesticide studies include some of the strongest experimentally treated or native/unknown-sample designs, and the additives/contaminants category offers broad matrix diversity but heterogeneous validation. Publication counts and low LODs therefore cannot serve as proxies for readiness in real-food analysis; the type of food validation, pretreatment burden, and agreement with reference methods are more discriminating indicators [17,29,39,54].

**Table 1:** Representative CD-based sensors for food chemical hazards.

Hazard Category	Target Analyte	CD System/Mode	LOD	Food Matrix	Recovery (%)	Ref.
Heavy metals/metalloids	Lead(II)	N-doped CDs; Cu nanocluster composite; ratiometric FL	0.0031 mg L^−1^	Laver	NR	[17]
Heavy metals/metalloids	Mercury(II)	N-doped CDs; FL	42.4 nM	Rice, grass carp, shrimp and crab	96.0–103.0%	[18]
Heavy metals/metalloids	Silver ion (Ag^+^)	N,S-co-doped CDs; paper-based; smartphone ratiometric FL	9.3 nM (solution); 75.79 nM (paper/phone)	Carp and river shrimp	95.01–105.76%	[19]
Heavy metals/metalloids	Lead(II) and silver(I)	CD platform; FL	Pb^2+^: 3.83 nM; Ag^+^: 8.2–9.0 nM	Carrot and potato	100–120%	[20]
Heavy metals/metalloids	Mercury(II)	Microwave-synthesized N-doped CDs derived from malonic acid and 2,3-diaminopyridine; FL	0.112 μmol L^−1^	Rice, shrimp, pear, orange, pineapple, cucumber and tomato	95.0–108.1%	[59]
Pesticides	Glyphosate	CD platform; FL	0.066 ng mL^−1^	Chinese chive	Leaf/stem/root: 107.2%, 89.3%, 101.9%	[29]
Pesticides	Acetamiprid	CD platform; aptamer-functionalized; FL	0.04 ng L^−1^ (buffer); 0.11 ng L^−1^ (lettuce)	Lettuce	Lettuce: 87–98%	[30]
Pesticides	Pendimethalin aclonifen benfluralin	CD platform; paper-based; smartphone FL	6.0–9.4 nM (solution probes); paper RGB NR	Tomato and lemon	95.0–105%	[33]
Pesticides	Carbendazim	CD platform; smartphone ratiometric FL	0.0209 μM (spectroscopic); 0.0700 μM (phone)	Apple, cucumber and melon	93.1–108.7%	[34]
Pesticides	Dichlorvos (DDVP)	N,S-co-doped CDs; MnO_2_-coupled system; ratiometric FL	1.2 μg L^−1^	Fruit juices; apple, celery, tomato, strawberry and okra	90.0–110.0%	[60]
Pesticides	Glyphosate	CD platform; electrochemical	2.02 ng mL^−1^	Tomato, cucumber, carrot, cauliflower, corn, soybean, chickpea and rice	90–102%	[61]
Veterinary drugs	Tetracyclines (TC, DC, OTC and CTC)	CD platform; FL	0.12–2.84 μM	Milk and chicken	95.37–110.4% (spiked); 74.0% (CRM)	[39]
Veterinary drugs	Tetracyclines (OTC, TC, CTC and DOX)	N,S-co-doped CDs; ratiometric FL	0.032 μM	Milk and honey	95.21–104.97%	[40]
Veterinary drugs	Tetracyclines	Zn-doped CDs; smartphone ratiometric FL	61.1 nM (solution); paper/phone: NR	Chicken, milk and egg	92.0–106.8%	[42]
Veterinary drugs	Sulfadiazine (SDZ)	CD platform; aptamer-functionalized; FL	0.97 ng mL^−1^ (0.0038 μM)	Honey	Honey: 93.2–103.2%	[43]
Veterinary drugs	Chloramphenicol (CAP)	CD platform; paper-based; smartphone ratiometric FL	0.03 μg L^−1^ (solution); paper/phone: NR	Milk, egg and honey	96.2–105.4%	[46]
Veterinary drugs	Norfloxacin	CD platform; FL	3.20 nM	Milk	94.60–97.00%	[62]
Veterinary drugs	Tetracycline (TET; OTC calibrated in standards only)	Zr,N-co-doped CDs; FL	TET: 0.029 μM; OTC: 0.016 μM	Cow, goat and camel milk	90.91–113.19%	[63]
Additives, adulterants, and processing contaminants	Acrylamide	CD platform; aptamer-functionalized; FL	Aqueous: 2.6 × 10^−7^ M; bread: 8.1 × 10^−7^ M	Bread	99.13–103.7%	[52]
Additives, adulterants, and processing contaminants	Allura Red	CD platform; FL	NR	Commercial beverages	100.25 ± 0.84%–102.50 ± 0.55%	[55]
Additives, adulterants, and processing contaminants	Curcumin (excess/unauthorized addition)	CD platform; ratiometric FL	0.089 μM	Chili, ginger and mustard powders	92.75–110.00%	[57]
Additives, adulterants, and processing contaminants	Melamine	AuNP/CD composite; portable fluorimeter; smartphone visual FL	3.6 nM (portable fluorimeter)	Milk	102.75–107.95%	[64]
Additives, adulterants, and processing contaminants	Nitrite	CD platform; smartphone FL/colorimetry	0.047 μg mL^−1^ (UV-vis); 0.014 μg mL^−1^ (FL); 0.16 μg mL^−1^ (phone color); 0.21 μg mL^−1^ (phone FL)	Luncheon meat, ham, sausage and pickled vegetables	83.3–106%	[65]
Additives, adulterants, and processing contaminants	Nitrite	CD platform; smartphone FL/colorimetry	0.06 μM (FL); 0.02 μM (solution color); 0.11 μM (phone RGB); 2.71 μM (textile RGB)	Luncheon meat, ham, spinach, pak choi and Chinese cabbage	94.52–103.40%	[66]

Note. Representative food-matrix studies are shown. Recoveries are reproduced as reported and do not, by themselves, establish method validity. AuNP, gold nanoparticle; CD, carbon dot; FL, fluorescence; LOD, limit of detection; NR, not reported; RGB, red-green-blue color space; UV-vis, ultraviolet–visible spectroscopy. Material terminology in this table is harmonized to the CD umbrella term; quantum-confinement behavior was not inferred when the original source did not provide supporting evidence.

## 4. Detection Applications in Real Food Matrices

Real food matrices represent a critical step in translating CD sensors from model systems to practical applications. High sensitivity and good linearity in standard solutions do not directly indicate analytical performance in foods [62,67], because proteins, lipids, natural pigments, salts, and other coexisting components may affect CD dispersion, optical responses, and target recognition. Evaluation of CD-based food sensors should therefore consider not only the LOD and linear range, but also food type, sample preparation, spike recovery, precision, and reference-method validation. The corresponding real-food evidence is summarized by sample type and validation approach in Table 2.

**Table 2:** Application of CD sensors in real food matrices.

Target	Food Matrix/Sample Type	Sample Preparation	Recovery/Validation	Main Limitation	Ref.
Lead(II)	Laver (U)	acid digestion → aqueous extraction → dilute → filter	RSD 1.9–4.5%; ICP-MS	Requires acid digestion	[17]
Silver ion (Ag^+^)	Carp and river shrimp (S)	NR; standard addition	95.01–105.76%; ICP-MS	Preparation details incompletely reported	[19]
Carbaryl	Apple (E)	homogenize → acetonitrile extraction → filter → incubate → concentrate → methanol extraction	NR; HPLC	Multi-step extraction	[28]
Glyphosate	Chinese chive (E + S)	homogenize → aqueous extraction → methanol extraction → centrifuge	89.3–107.2%; HPLC-MS	Limited matrix diversity	[29]
Acetamiprid	Lettuce (U + S)	homogenize → methanol extraction → centrifuge → filter → concentrate → reconstitute	87–98%; LC-MS/MS	Multi-step extraction	[30]
Tetracyclines (TC, DC, OTC and CTC)	Milk and chicken (CRM + S)	deproteinize → homogenize → sonicate → centrifuge	95.37–110.4% (spiked); 74.0% (CRM)	CRM result showed incomplete analyte recovery	[39]
Sulfadiazine (SDZ)	Honey (S)	ethyl acetate extraction → centrifuge → concentrate → dilute	93.2–103.2%; HPLC-MS	Mostly spiked validation	[43]
Acrylamide	Bread (S)	QuEChERS	99.13–103.7%; LC-MS/MS	Mostly spiked validation	[52]
New Coccine	Beverages, confectionery and chili powder (U + S)	filter → dilute	97.5–104.0%; HPLC	Commercial samples plus spiking; no incurred/CRM validation	[58]
Mercury(II)	Rice, shrimp, pear, orange, pineapple, cucumber and tomato (S)	homogenize → dilute → incubate → centrifuge → filter	95.0–108.1%; RSD ≤ 1.4%; ICP-MS	Multi-step extraction	[59]
Dichlorvos (DDVP)	Fruit juices; apple, celery, tomato, strawberry and okra (U + S)	filter → homogenize → acetonitrile extraction → centrifuge → cleanup	90.0–110.0%; GC-MS	Multi-step extraction	[60]
Glyphosate	Tomato, cucumber, carrot, cauliflower, corn, soybean, chickpea and rice (U + S)	PBS/methanol extraction → vortex → centrifuge → PBS dilution → filtration	90–102%; GC-MS/HPLC	Multi-step extraction	[61]
Nitrite	Luncheon meat, ham, sausage and pickled vegetables (U + S)	cleanup → sonicate → incubate → ethanol extract → vortex → dilute	83.3–106%; reference colorimetry	Color interference requires cleanup	[65]
Nitrite	Luncheon meat, ham, spinach, pak choi and Chinese cabbage (U + S)	homogenize → aqueous extraction → sonicate → centrifuge → filter	94.52–103.40%; reference colorimetry	Multi-step extraction	[66]
Organophosphate pesticides	Apple and peach (U + S)	homogenize → centrifuge → dilute	99–101%; GC-MS	Limited to two fruit matrices	[68]
Tartrazine	Beverages, confectionery and lemon (U + S)	aqueous extraction → filter	92.7–105%; GB 4481.1-2010	Commercial samples plus spiking; no incurred/CRM validation	[69]
Ciprofloxacin	Milk and egg (S)	sonicate → acetonitrile extraction → centrifuge → concentrate → dilute	33.09–115.07%; no reference method	Poor recovery in part of the tested matrices	[70]
Dichlorvos	Chinese cabbage, lettuce and spinach (E + S)	homogenize → acetone extract → centrifuge	90.56–107.0%; HPLC-MS	Leafy-vegetable scope only	[71]

Note. Sample-type codes: S, spiked food; U, commercial food analyzed at native or unknown concentration; E, experimentally contaminated or incurred sample; CRM, certified reference material. GC-MS, gas chromatography–mass spectrometry; HPLC, high-performance liquid chromatography; HPLC-MS, high-performance liquid chromatography–mass spectrometry; ICP-MS, inductively coupled plasma mass spectrometry; LC-MS/MS, liquid chromatography–tandem mass spectrometry; NR, not reported; QuEChERS, quick, easy, cheap, effective, rugged, and safe; RSD, relative standard deviation.

Among the 106 studies in the broad analytical corpus, not all experiments labeled as “real sample” involved actual foods. Studies validated only in standard solutions, water, or non-food matrices—including the reports represented by Refs. [13,14,36]—were retained solely as contextual sensor evidence and were excluded from the 68-study food-matrix subset, all real-food statistics, and Table 1, Table 2 and Table 3. Most food methods still reported homogenization, centrifugation, filtration, deproteinization, organic-solvent extraction, or acid digestion, demonstrating that a rapid CD sensor response does not necessarily mean that the complete food-analysis workflow is equally rapid. The ciprofloxacin study in milk and egg illustrates this limitation: although several conditions yielded recoveries near 90–115%, egg analysis by SWV with the CPE/ZnO/CD electrode produced only 33.09–47.56% recovery [70], indicating pronounced method- and matrix-dependent bias.

### 4.1. Types of Real Food Samples and Current Applications

As shown in Figure 2, CD-based food detection exhibits clear coupling between hazard categories and food matrices. The 68 real-food studies yielded 98 study–food matrix combinations. A study involving multiple food categories was counted once in each category; therefore, these 98 combinations do not represent 98 independent studies. Veterinary-drug research was concentrated mainly on dairy products, pesticide research predominantly addressed fruits and vegetables, heavy-metal food applications were fewer but relatively concentrated on aquatic products, and the matrix distribution of additives, adulterants, and processing contaminants was more dispersed [72,73,74].

Across the literature, dairy products contributed 24 study–food matrix combinations, 20 of which came from veterinary-drug and antibiotic studies, making this the most prominent hazard-matrix pairing. Several studies expanded beyond milk alone to include milk, eggs, chicken, honey, and other animal-derived foods [42,75,76]. In terms of matrix coverage, Lu et al. further validated the same tetracycline sensing system in cow, goat, and camel milk, directly comparing matrices within the same broad food category [63].

Fruits and vegetables contributed 28 study–food matrix combinations, 17 of which came from pesticide studies. Apples, tomatoes, lettuce, Chinese chives, cucumbers, carrots, and other foods were used for method validation, and the evidence covered a range of spiked real-food samples [77,78,79,80]. More notably, Peng et al. used experimentally treated Chinese chives for tissue- and time-resolved residue monitoring [29], whereas Jiao et al. first determined the unknown native level in commercial lettuce [30]. Both designs more closely approximate practical pesticide-residue monitoring than simple post-extraction spiking.

Aquatic products contributed only six study–food matrix combinations, four of which came from heavy-metal studies involving fish, shrimp, and crab [19,59]. This distribution supports the conclusion that heavy-metal food applications are relatively concentrated on aquatic products, while also showing that the overall evidence base is substantially smaller than that for dairy products-veterinary drugs and fruits and vegetables-pesticides.

Additives, adulterants, and processing contaminants generated 26 study–food matrix combinations distributed across dairy products, meat products, fruits and vegetables, beverages, confectionery, spices, and other processed foods [69,81,82]. Thus, inclusion of a “real food” does not imply a uniform validation approach: spiked real-food samples, commercial samples at native or unknown concentrations, naturally contaminated or incurred samples, and certified reference materials should be reported separately rather than grouped indiscriminately as “real samples.”

### 4.2. Sample Preparation and Matrix Interference

As shown in Figure 3, the sample-preparation steps reported by the 68 real-food studies were not mutually exclusive. Centrifugation and filtration were reported in 39 and 35 studies, respectively; organic-solvent extraction, homogenization/grinding, sonication, and deproteinization/defatting were also common, whereas direct analysis or minimal pretreatment was reported in only a few studies [83,84,85]. The figure includes only operations explicitly reported in the articles, and missing information was not interpreted as the absence of pretreatment. These operations can improve apparent sensitivity and recovery by removing pigments, proteins, lipids, particulates, and other quenchers, or by concentrating the analyte before measurement. However, these gains are obtained at the cost of longer analysis time, greater solvent and reagent consumption, additional benchtop equipment, and operator-dependent variability. Consequently, a low instrumental LOD in a purified standard or extract does not necessarily translate into a faster or less costly end-to-end food analysis.

Different hazards and food types exhibited different combinations of sample-preparation procedures. Pesticide analysis commonly involved homogenization, organic-solvent extraction, and centrifugation/filtration, with sonication, concentration, or reconstitution used in some methods [86]. Dairy products and other animal-derived foods usually required deproteinization, defatting, centrifugation, and filtration, and some methods additionally employed acetonitrile extraction and rotary evaporation [70]. For example, Wei et al. used QuEChERS treatment for acrylamide analysis in bread [54], whereas Li et al. required digestion, dilution, and filtration before CD-based Pb^2+^ measurement in laver [17].

In addition to sample-processing operations, natural food components may produce substantial matrix effects. Dark-colored beverages, fruit juices, tea, and spices can exhibit strong absorption or natural fluorescence and may interfere with CD optical signals. Protein- and lipid-rich foods can promote probe adsorption or aggregation, while high salt concentrations and differences in pH may alter CD surface states [7,56,57]. Consequently, assessment of the “rapidity” of a CD method must consider the complete analytical workflow: rapid sensing does not necessarily translate into rapid end-to-end food analysis.

Future method development should therefore incorporate sample processing into portable-system design. Simple dilution, single-step filtration, paper-based extraction, or microfluidic cleanup should be used to minimize reliance on large laboratory instruments and multistep operations wherever possible [31,60,87].

### 4.3. Analytical Performance and Validation Approaches in Real Foods

Many CD-based food-detection studies reported recoveries of approximately 90–110% in spiked samples, and some also reported low RSD values. Liu et al. obtained Hg^2+^ recoveries of approximately 95.0–108.1% with RSD ≤ 1.4% in rice, shrimp, and several fruits and vegetables, with confirmation by ICP-MS [59]. Other studies of Hg^2+^ in aquatic products and grains reported similar spiking performance [18]. For processed foods, Song et al. reported nitrite spike validation in ham, cured pork, and salted duck eggs [88].

However, spike recovery alone cannot fully represent analytical reliability in real foods. Naturally present or incurred analytes may bind to proteins, tissues, or other components, and their extraction efficiency and response behavior may differ from those of standards added after sample preparation. The study by Lin et al. provides representative evidence: results from conventional spiked samples were satisfactory, whereas recovery of one analyte in a certified reference material for tetracyclines in chicken was approximately 74%, indicating that incomplete extraction or matrix effects may persist under incurred-contamination conditions [39]. Other milk and egg studies likewise demonstrate that spiking results alone cannot encompass all uncertainties in actual samples [89,90].

Validation against reference methods is also important. Jiao et al. used LC-MS/MS to validate acetamiprid measurements in commercial lettuce [30]; Li et al. used ICP-MS to confirm Pb^2+^ analysis in laver [17]; and Wei et al. compared acrylamide results in bread with LC-MS/MS [54]. Same-sample comparisons of this kind provide stronger external validation than spike recovery alone.

Applicability cannot be extrapolated directly across food matrices. Even within dairy products, fruits and vegetables, or meat, samples may differ substantially in fat, protein, pigments, acidity, and salt content. CD platforms with greater application value should be validated in multiple and compositionally distinct food matrices [91,92,93,94], while clearly distinguishing spiked real-food samples from commercial samples at native or unknown concentrations and naturally contaminated or incurred samples.

Overall, CD sensors have been applied relatively broadly to real food matrices; however, the current evidence is characterized by uneven matrix coverage, a high proportion of spiked samples, and insufficient use of commercial samples at native or unknown concentrations, naturally contaminated or incurred samples, and reference-method validation [27,49,95,96]. The next stage should move from demonstrating that CDs can detect a target in food to demonstrating that they can quantify it in complex real foods with stability, accuracy, and reproducibility.

## 5. Portable and On-Site Detection

As shown in Figure 4, portable CD-based detection of food chemical hazards can be represented as a continuous process comprising real food sampling, matrix pretreatment, a portable CD platform, signal acquisition, calibration-based quantification, and field application. Complex food samples usually require pretreatment such as homogenization, extraction or deproteinization, centrifugation, and filtration to reduce interference from proteins, lipids, particulate matter, and other coexisting components. CD detection can then be configured as a paper-based platform, hydrogel/solid-state platform, or solution-based portable-imaging platform [32]. Target-induced changes in color, fluorescence, or thermal signals generate detectable responses, while smartphones, portable optical devices, or thermal-imaging devices capture digital signals such as RGB or HSV values, fluorescence intensity, color-channel ratios, or temperature changes [65,97]. Calibration curves and appropriate validation then convert the raw optical or thermal response into analyte concentration, enabling a transition from simple visualization to quantitative analysis. Ultimately, portable CD systems may support rapid screening, on-site quantification, and standardized and repeatable food-safety monitoring. It should be emphasized that portable visualization alone does not constitute validated quantitative analysis; genuine field application also depends on simplified pretreatment, standardized signal acquisition, and the reliability and reproducibility of calibration models. Table 3 summarizes representative studies of portable CD-based food detection.

**Table 3:** Representative portable CD-based platforms for food chemical hazard detection.

Target	Food Matrix	Portable Platform	Readout/Quantification	LOD	Reference Validation	Ref.
Silver ion (Ag^+^)	Carp and river shrimp	Paper strip + smartphone	B/R; calibration; dual-phone	75.79 nM	ICP-MS	[19]
Thiram and paraquat	Lotus seed, coix seed and ginseng	Portable imaging box + smartphone	RGB; calibration	NR (solution: 7.49 nM for thiram; 3.03 nM for paraquat)	NR	[32]
Pendimethalin, aclonifen and benfluralin	Tomato and lemon	Paper strip + smartphone	RGB; calibration	NR (solution: 6.0–9.4 nM)	GC-MS	[33]
Carbendazim	Apple, cucumber and melon	Portable imaging box + smartphone	B/R; calibration; cross-device	0.0700 μM	NR	[34]
Tetracyclines	Chicken, milk and egg	Paper strip + smartphone	G/B; semi-quantitative calibration	NR (solution: 61.1 nM)	HPLC	[42]
Chloramphenicol (CAP)	Milk, egg and honey	Paper strip + smartphone	B/G; calibration	NR (solution: 0.03 μg L^−1^)	NR	[46]
Melamine	Milk	96-well imaging + portable fluorimeter	FL intensity; calibration	3.6 nM	HPLC-DAD	[64]
Nitrite	Luncheon meat, ham, sausage and pickled vegetables	Portable imaging box + smartphone	Color/FL dual mode; calibration	0.16 μg mL^−1^ (phone color); 0.21 μg mL^−1^ (phone FL)	Reference colorimetry	[65]
Dichlorvos	Chinese cabbage, lettuce and spinach	Hydrogel + thermal camera	Photothermal ΔT; calibration	4.85 μg L^−1^	HPLC-MS	[71]
Tetracyclines	Milk	Portable imaging box + smartphone	RGB; ratiometric calibration	0.069 μM	HPLC	[76]
Nitrite	Ham, cured pork and salted duck egg	Hydrogel + smartphone	R/G; calibration	1.80 μM (phone); 1.78 μM (hydrogel)	Reference colorimetry	[88]
Gentamicin	Milk and egg	Portable imaging box + smartphone	RGB and G/R; calibration	0.434 μM (smartphone color); 0.587 μM (smartphone FL)	NR	[92]

Note. Only platforms with calibration-based quantitative or semi-quantitative portable readouts were retained. Portable-specific LODs are shown when available; otherwise, the solution LOD is identified in parentheses. B/R, B/G, G/B, G/R, and R/G denote ratios between the indicated red, green, and blue channels. FL, fluorescence; GC-MS, gas chromatography–mass spectrometry; HPLC, high-performance liquid chromatography; HPLC-DAD, high-performance liquid chromatography with diode-array detection; HPLC-MS, high-performance liquid chromatography–mass spectrometry; ICP-MS, inductively coupled plasma mass spectrometry; LOD, limit of detection; NR, not reported; RGB, red-green-blue color space; ΔT, temperature change.

### 5.1. Solid-State and Portable Sensing Platforms

Paper-based platforms generally immobilize CDs on filter paper, cellulose paper, or paper strips, thereby reducing liquid transfer and creating storable reaction zones. Food studies have applied such systems to targets including tetracyclines and propiconazole [42,86]. Paper-based or thin-film platforms reported by Rizvi et al. [98], Kundu et al. [99], and Vyas and Joshi [100] were validated only in standard systems, pharmaceutical preparations, or non-food water samples; here, they are included solely as design references with potential for translation to food analysis, rather than as evidence of food application.

In food applications, hydrogels, polymer films, textiles, and other solid supports can provide confined reaction environments and improve operational stability. Song et al. immobilized the nitrite response for food analysis in an agarose hydrogel [88]. Zhang et al. coated cotton fabric with a CD gel to produce cuttable strips for nitrite analysis in pre-processed vegetables [66]. Yang et al. used an agarose hydrogel to detect dichlorvos in leafy vegetables through color and thermal readouts [71]. These studies indicate that the central value of solid-state food platforms lies in integrating the reaction interface, support, and readout conditions into a reproducible operational unit.

Solid-state formats may also introduce uneven CD loading, variation in membrane thickness or porosity, effects of drying conditions, carrier-to-carrier variation, and insufficient storage stability. Transferring a platform from solution to paper, hydrogel, or film therefore does not automatically ensure reliable field analysis; material fabrication and batch-to-batch quality control must still be standardized. Device-fabrication and stability issues identified in non-food platforms, including films for water samples and vapor-phase quartz-crystal microbalance systems [101,102,103], are used here only as supplementary translational context.

### 5.2. Smartphone-Assisted Quantitative Detection

Smartphones can extract RGB and HSV values and color-channel ratios such as B/R, G/B, and R/G from paper, hydrogel, solution, or portable-imaging-box formats [32]. In this way, changes in fluorescence, color, or thermal images can be converted into continuous numerical values and analyte concentrations predicted using calibration curves [64,65]. Smartphone photography alone is only image recording; quantitative analysis requires an established and validated concentration-signal relationship.

Smartphone quantification has been extended to pesticides, veterinary drugs, nitrite, melamine, colorants, and other targets. Han et al. established a carbendazim calibration model based on the B/R signal [34]. Xu et al. integrated an Ag^+^ paper strip, a 3D-printed dark box, and smartphone readout into a single quantitative platform [19]. Peng et al. used an Android smartphone and PhotoMetrix for calibration-based quantification of nitrite in commercial meat products and pickled vegetables [65]. In contrast, the dual-mode sensor developed by Liang et al. for malathion in cabbage emphasized visual readout [104], illustrating that an assay that can be photographed or observed is not necessarily a smartphone-based quantitative method.

Recent studies have begun to compare consistency among different smartphones. Han et al. compared carbendazim B/R readings obtained with iPhone, Xiaomi, Huawei, and other devices [34], while Xu et al. also performed cross-phone validation of an Ag^+^ paper-based platform [19]. Such designs directly reveal potential systematic biases arising from differences in cameras, automatic exposure, white balance, and image-processing algorithms [97] and represent an important step toward transferable smartphone quantification. Fixed-geometry imaging chambers, including 3D-printed dark boxes equipped with constant-intensity LEDs and a controlled camera-sample distance, can suppress ambient-light and positioning variability and improve normalization of RGB/HSV or channel-ratio measurements [19,34]. Nevertheless, such enclosures do not fully eliminate device-specific differences in camera sensors, automatic exposure, white balance, and image processing; cross-device calibration or transfer correction is still required [97].

The key requirement for smartphone-based detection is therefore not merely the ability to obtain a reading, but the use of standardized illumination, fixed imaging distance, color-correction references, and cross-device calibration [6,8,97] so that comparable quantitative results can be obtained across phones, operators, and time points.

### 5.3. From Visual Detection to On-Site Quantification

Portable CD detection can be divided into three levels: visual observation of fluorescence or color changes, smartphone-assisted semi-quantification, and quantitative analysis based on calibration models [33,42,99,105]. For practical food analysis, a method approaches genuine on-site quantification only when a defined relationship between concentration and fluorescence intensity, color-channel ratio, temperature, or another signal has been established and validated. Visualization and quantification should not be conflated.

Representative studies have used ratio signals such as G/B, B/R, and R/G or temperature changes in thermal images for quantification [66,71,88,106]. Ratiometric signals offer a degree of internal correction and can reduce the influence of illumination changes and variations in probe concentration; however, quantitative maturity still depends on the calibration range, validation with independent samples, cross-device consistency, and comparison with reference methods.

Establishing a calibration curve does not mean that a method is ready for field application [107,108,109]. Genuine on-site quantification also requires reproducible sample processing, carriers or devices, and imaging conditions.

Overall, the technical pathway in Figure 4 and the representative cases in Table 3 [19,65,66] jointly indicate that CD-based food detection is progressing from visual observation toward calibration-based portable quantification. Specific issues of standardization and real-world validation are discussed in Section 6.

## 6. Challenges and Future Directions

Although CD sensors have demonstrated high sensitivity and substantial potential for portable detection of food chemical hazards, practical applications remain constrained by complex food matrices, sample preparation, method reproducibility, and field standardization [4,110]. Future research should shift gradually from the sole pursuit of lower detection limits toward reliable detection in real foods and optimization of complete analytical workflows.

### 6.1. From Lower Detection Limits to Greater Analytical Reliability

Many current CD sensing studies still use the LOD and linear range as their principal performance indicators, and some model systems have achieved nanomolar or even lower levels [101,111,112,113]. For systems that have already reached or fallen substantially below concentration ranges relevant to practical regulation, further reductions in the LOD may provide limited marginal application value. Accuracy, precision, reproducibility, resistance to interference, batch-to-batch consistency, recovery in real foods, and agreement with reference methods more directly determine practical value.

Future studies should strengthen reproducibility assessments across CD batches, days, and operators and systematically examine material-storage stability and quantitative accuracy in real foods. Paper-based, hydrogel, and portable-imaging platforms [38,96,97,114] should additionally be evaluated for consistency across carrier batches, readout devices, and environmental conditions.

### 6.2. Improving Compatibility with Complex Food Matrices and Simplifying Sample Preparation

Proteins, lipids, natural pigments, salts, and other coexisting components in real foods may substantially affect CD optical responses; therefore, excellent performance in standard solutions does not directly represent analytical performance in real foods. As shown above, dairy products often require deproteinization and defatting, pesticide analysis commonly involves organic-solvent extraction, centrifugation, and filtration, and some heavy-metal analyses in foods still depend on acid digestion [7,17]. By contrast, highly sensitive CD probes for mercury ions [115,116] demonstrate probe performance but do not provide direct evidence of acid-digestion requirements for food analysis.

Future CD sensor development should therefore place greater emphasis on matrix-oriented design-that is, optimizing probe-surface properties and signal modes according to the composition of the target food-while integrating sample processing with detection [31,106,117]. Simple dilution, membrane filtration, paper-based extraction, or microfluidic cleanup should be used to reduce complex laboratory operations. Truly rapid detection should be reflected in simplification of the complete analytical workflow, rather than only in rapid sensor response.

### 6.3. From Single-Analyte Detection to Multi-Analyte and Intelligent Analysis

Existing CD-based food-sensing studies still focus mainly on single-analyte detection, whereas several pesticides, veterinary drugs, or other hazards may coexist in foods. Future systems could use multichannel-emission CDs, sensor arrays, and multimodal signals [45,118] to construct multidimensional responses and combine them with chemometrics or machine learning [119] for multi-analyte classification and quantification.

This direction may improve the efficiency of CD platforms for rapid food screening, but intelligent algorithms must be based on reliable experimental data. Future studies should validate model performance with independent food samples, report the division of training and test sets, and avoid emphasizing high classification accuracy from limited data while overlooking model transferability.

### 6.4. Standardizing Portable Detection and Strengthening Real-World Validation

Paper-based, hydrogel, thin-film, and smartphone platforms have substantially advanced CD detection toward field applications, but existing studies lack uniform standards for illumination, smartphone model, imaging distance, image-processing method, and calibration procedure. Differences in cameras and automatic image-processing algorithms may cause substantial signal variation; consequently, “smartphone-readable” does not mean “reliable on-site quantification” [6,8,97]. In addition to optical imaging, electrochemical platforms based on CD composites have been used for bisphenol A detection [120]; their field translation likewise requires standardization of electrode preparation, calibration procedures, and batch-to-batch consistency.

Future studies should establish standardized imaging environments and calibration methods and strengthen validation across devices, operators, and long-term storage conditions. They should also increase the use of commercial samples at native or unknown concentrations, naturally contaminated or incurred samples, and certified reference materials, with same-sample comparisons against reference methods such as HPLC, LC-MS/MS, GC-MS, or ICP-MS [12,39,89]. CD sensors can progress from laboratory proof of concept to on-site food analysis only by integrating real-food evidence, simplified pretreatment, stable materials, and standardized digital readout.

Overall, the next stage of CD-based detection of food chemical hazards should move from lower LODs to greater reliability, from standard solutions to explicitly stratified real-food evidence, from single-analyte detection to multi-analyte analysis, and from portable demonstration to standardized on-site quantification [1,2].

This transition requires sensing-material performance, end-to-end sample processing, agreement with reference methods, and reproducibility under real-world conditions to be evaluated as components of a single analytical workflow [4,110].

## 7. Conclusions

CD-based sensing of food-safety-relevant analytes has expanded across heavy metals and metalloids, pesticides, veterinary drugs and antibiotics, food additives, adulterants, and processing contaminants. The two-tier evidence analysis clarifies that the 106-study broad analytical corpus does not represent 106 food applications: only 68 studies experimentally evaluated an actual food matrix. Across those food-matrix studies, translational maturity was uneven—veterinary-drug work contributed the densest evidence in dairy products, pesticide studies were concentrated in fruits and vegetables and included several stronger incurred or native/unknown-sample designs, heavy-metal applications were comparatively limited and focused on aquatic products, and the additives/contaminants category combined broad matrix coverage with heterogeneous validation. These differences show why publication counts and low LODs alone cannot provide a complete picture of practical readiness.

Paper-based, hydrogel, portable-imaging, and smartphone platforms are advancing CD detection from visual observation toward calibration-based digital quantification, but cross-device consistency, carrier reproducibility, complex sample preparation, and field standardization still constrain practical deployment. Future CD-based food detection should therefore shift from the sole pursuit of lower detection limits toward greater analytical reliability, simpler sample processing, stronger compatibility with complex matrices, multi-analyte detection, and standardized on-site quantification. Only by evaluating sensing materials, food-matrix validation, pretreatment workflows, reference-method agreement, and portable readout as one analytical system can CD technology progress toward reproducible food-safety monitoring.

## Data Availability

No new data were created or analyzed in this study. Data sharing is not applicable to this article.

## References

[1] Luo X., Han Y., Chen X., Tang W., Yue T., Li Z. (2020). Carbon dots derived fluorescent nanosensors as versatile tools for food quality and safety assessment: A review. Trends Food Sci. Technol..

[2] Zhang W., Zhong H., Zhao P., Shen A., Li H., Liu X. (2022). Carbon quantum dot fluorescent probes for food safety detection: Progress, opportunities and challenges. Food Control.

[3] Yu Y., Zhang L., Gao X., Feng Y., Wang H., Lei C., Yan Y., Liu S. (2022). Research progress in the synthesis of carbon dots and their application in food analysis. Biosensors.

[4] Singh H., Saima, Aggarwal V., Kachore A., Bala E., Kumar R., Sharma R.K., Verma P.K. (2025). Carbon dots: An emerging food analysis nanoprobes for detection of contaminants. Food Chem..

[5] Sivakumar R., Lee N.Y. (2021). Recent progress in smartphone-based techniques for food safety and the detection of heavy metal ions in environmental water. Chemosphere.

[6] Nelis J.L.D., Tsagkaris A.S., Dillon M.J., Hajslova J., Elliott C.T. (2020). Smartphone-based optical assays in the food safety field. TrAC Trends Anal. Chem..

[7] Wu Q., Deng Z., Duan N., Wang Z., Wu S. (2026). Tackling food sample matrix effects for precise food safety detection: Novel strategies and application progress. Trends Food Sci. Technol..

[8] Gopal J., Muthu M. (2024). Handheld portable analytics for food fraud detection, the evolution of next-generation smartphone-based food sensors: The journey, the milestones, the challenges debarring the destination. TrAC Trends Anal. Chem..

[9] Page M.J., McKenzie J.E., Bossuyt P.M., Boutron I., Hoffmann T.C., Mulrow C.D., Shamseer L., Tetzlaff J.M., Akl E.A., Brennan S.E. (2021). The PRISMA 2020 statement: An updated guideline for reporting systematic reviews. BMJ.

[10] Rethlefsen M.L., Kirtley S., Waffenschmidt S., Ayala A.P., Moher D., Page M.J., Koffel J.B., PRISMA-S Group (2021). PRISMA-S: An extension to the PRISMA statement for reporting literature searches in systematic reviews. Syst. Rev..

[11] White H., Albers B., Gaarder M., Kornør H., Littell J., Marshall Z., Matthew C., Pigott T., Snilstveit B., Waddington H. (2020). Guidance for producing a Campbell evidence and gap map. Campbell Syst. Rev..

[12] Nguyen D.H.H., El-Ramady H., Prokisch J. (2025). Food safety aspects of carbon dots: A review. Environ. Chem. Lett..

[13] Aygun A., Cobas I., Tiri R.N.E., Sen F. (2024). Hydrothermal synthesis of B, S, and N-doped carbon quantum dots for colorimetric sensing of heavy metal ions. RSC Adv..

[14] Cheng Z., Du F., Sun L., Jiang L., Ruan G., Li J. (2019). Nitrogen-doped carbon quantum dots as a “turn-off” fluorescent probes for highly selective and sensitive detection of mercury(II) ions. ChemistrySelect.

[15] Gontrani L., Bauer E.M., Nucara A., Tagliatesta P., Carbone M. (2022). Highly specific silver ion detection by fluorescent carbon quantum dots. Chemosensors.

[16] Yang J., Guo Z., Yue X. (2021). Preparation of carbon quantum dots from corn straw and their application in Cu^2+^ detection. BioResources.

[17] Li W., Hu X., Li Q., Shi Y., Zhai X., Xu Y., Li Z., Huang X., Wang X., Shi J. (2020). Copper nanoclusters @ nitrogen-doped carbon quantum dots-based ratiometric fluorescence probe for lead (II) ions detection in porphyra. Food Chem..

[18] Shan D., Yu H., Yang Z., Li H., Jia R., Zhang Y. (2025). Nitrogen-doped carbon quantum dots (NCQDs) detected to mercury ions in food monitoring. Food Chem..

[19] Xu D., Chen S., Zhou L., Zhang F., Yang H., Cao L., Tan R., Chai F. (2026). Orange-emissive nitrogen doped carbon dots as fluorescent for rapid sensing Ag^+^ in food water and soil. J. Environ. Chem. Eng..

[20] Javeria H., Abbas M.Q., Chen S.H., Keshat B.E., Du Z. (2025). Scalability of sulfur-functionalized carbon quantum dots from peanut shells: A sustainable sensor of high colorimetric heavy metal detection. J. Environ. Chem. Eng..

[21] Liu Q., Gao X., Liu Z., Gai L., Yan Y., Ma H. (2023). Sensitive and selective electrochemical detection of lead(II) based on waste-biomass-derived carbon quantum dots@zeolitic imidazolate framework-8. Materials.

[22] Bahtiar A., Saputra R.N., Setianto (2022). Physical and optical properties of N,S-codoped carbon quantum dots derived from cigarette butts for mercury ions detection. J. Phys. Conf. Ser..

[23] Wu S., Yin Y., Sun C., Song W. (2022). Efficient synthesis of highly photo-stable N-doped carbon quantum dots and their applications in detection and cellular imaging of mercury ions. ChemistrySelect.

[24] Chen Y., Qin X., Yuan C., Shi R., Wang Y. (2020). Double responsive analysis of carbaryl pesticide based on carbon quantum dots and Au nanoparticles. Dyes Pigments.

[25] Cheng W., Zheng Z., Yang J., Chen M., Yao Q., Chen Y., Gao W. (2019). The visible light-driven and self-powered photoelectrochemical biosensor for organophosphate pesticides detection based on nitrogen doped carbon quantum dots for the signal amplification. Electrochim. Acta.

[26] Wu X., Song Y., Yan X., Zhu C., Ma Y., Du D., Lin Y. (2017). Carbon quantum dots as fluorescence resonance energy transfer sensors for organophosphate pesticides determination. Biosens. Bioelectron..

[27] Mahmoudi N., Fatemi F., Rahmandoust M., Mirzajani F., Ranaei Siadat S.O. (2023). Development of a carbon quantum dot-based sensor for the detection of acetylcholinesterase and the organophosphate pesticide. Heliyon.

[28] Li H., Sun C., Vijayaraghavan R., Zhou F., Zhang X., MacFarlane D.R. (2016). Long lifetime photoluminescence in N, S co-doped carbon quantum dots from an ionic liquid and their applications in ultrasensitive detection of pesticides. Carbon.

[29] Peng Z., Zeng M., Wu S., Yan Z., Rui J., Qiu P., Wang X. (2022). Development of a pH-responsive, SO_4_^2−^-loaded Fe and N co-doped carbon quantum dots-based fluorescent method for highly sensitive detection of glyphosate. Anal. Chim. Acta.

[30] Jiao Z., Zhang H., Jiao S., Guo Z., Zhu D., Zhao X. (2019). A turn-on biosensor-based aptamer-mediated carbon quantum dots nanoaggregate for acetamiprid detection in complex samples. Food Anal. Methods.

[31] Li Z., Pu H., Wei Q. (2024). Ti_3_C_2_T_x_ MXene-based fluorescent aptasensor for detection of dimethoate pesticide. Biosensors.

[32] Chen L., Lu J., Luo M., Yu H., Chen X., Deng J., Hou X., Hao E., Wei J., Li P. (2022). A ratiometric fluorescent sensing system for the selective and ultrasensitive detection of pesticide residues via the synergetic effects of copper nanoclusters and carbon quantum dots. Food Chem..

[33] Öztürk D., Sanko V., Ömeroğlu İ., Khataee A., Demirbaş E., Durmuş M., Yeşilot S., Kılıç Z., Demirbaş Ü., Şenocak A. (2026). Detection of herbicides in food samples using hybrid carbon dot sensors and test kit fabrication via paper-based analytical devices. Microchem. J..

[34] Han Y., Luo X., Li C., Ma Y., Jiang C., Shang F., Li Z., Wang H. (2026). Dual-emission carbon dots hybrid for ratiometric fluorescence and visual on-site detection of carbendazim residues in fruits and vegetables. J. Food Process Eng..

[35] Liu M.L., Chen B.B., Yang T., Wang J., Liu X.D., Huang C.Z. (2017). One-pot carbonization synthesis of europium-doped carbon quantum dots for highly selective detection of tetracycline. Methods Appl. Fluoresc..

[36] Wei X., Lv L., Zhang Z., Guan W. (2020). Preparation of molecularly imprinted fluorescence sensor based on carbon quantum dots via precipitation polymerization for fluorescence detection of tetracycline. J. Appl. Polym. Sci..

[37] Wang C., Ma Z., Yao Z., Li P., Zhang J., Zhang W., Zhao X., Shi H. (2025). High-sensitivity detection of tetracycline using phosphorus-doped carbon quantum dots prepared by pyrolysis method as fluorescent probes. Phys. Scr..

[38] Zhang M., Dong X., Ji G., Wang J., Wang T., Liu Q., Niu Q. (2025). Synergistic effect of 2D covalent organic frameworks confined 0D carbon quantum dots film: Toward molecularly imprinted cathodic photoelectrochemical platform for detection of tetracycline. Biosens. Bioelectron..

[39] Lin X., Wang J., Xia M., Li S., Xu Y., Sun J., Leng H., Song T., Xing Y., Yang M. (2026). A dual-functional fluorescent sensor based on a single soybean-derived carbon dots for discrimination and quantification of tetracyclines in food. Food Chem..

[40] Fan Y., Shen L., Liu Y., Hu Y., Long W., Fu H., She Y. (2023). A sensitized ratiometric fluorescence probe based on N/S doped carbon dots and mercaptoacetic acid capped CdTe quantum dots for the highly selective detection of multiple tetracycline antibiotics in food. Food Chem..

[41] Fan Y., Qiao W., Long W., Chen H., Fu H., Zhou C., She Y. (2022). Detection of tetracycline antibiotics using fluorescent “turn-off” sensor based on S, N-doped carbon quantum dots. Spectrochim. Acta A Mol. Biomol. Spectrosc..

[42] Hu F., Fu Q., Li Y., Yan C., Xiao D., Ju P., Hu Z., Li H., Ai S. (2024). Zinc-doped carbon quantum dots-based ratiometric fluorescence probe for rapid, specific, and visual determination of tetracycline hydrochloride. Food Chem..

[43] Zhang L., Feng Z., Zhou Y., Wu Y., Le T. (2022). A highly sensitive and selective “on–off–on” fluorescent aptamer sensor based on tea residue carbon quantum dots for the detection of sulfadiazine in honey. New J. Chem..

[44] Du H., He L., Zhang M., Manyande A., Chen H. (2023). Carbon quantum dots derived from fish scales as fluorescence sensors for detection of malachite green. J. Food Meas. Charact..

[45] Sajwan R.K., Solanki P.R. (2023). Gold@Carbon quantum dots nanocomposites based two-in-one sensor: A novel approach for sensitive detection of aminoglycosides antibiotics in food samples. Food Chem..

[46] Yang X., Hou X., Wang L., Hou L., Han Y., Li M., Ding L., Sun X. (2026). PET-featured fluorescent test papers with smartphone platform based on chitosan carbon dots for visual detecting chloramphenicol in animal-derived foods. Microchem. J..

[47] Su A., Zhong Q., Chen Y., Wang Y. (2018). Preparation of carbon quantum dots from cigarette filters and its application for fluorescence detection of Sudan I. Anal. Chim. Acta.

[48] Feng Z., Li Z., Zhang X., Shi Y., Zhou N. (2017). Nitrogen-doped carbon quantum dots as fluorescent probes for sensitive and selective detection of nitrite. Molecules.

[49] Wu X., Wu L., Cao X., Li Y., Liu A., Liu S. (2018). Nitrogen-doped carbon quantum dots for fluorescence detection of Cu^2+^ and electrochemical monitoring of bisphenol A. RSC Adv..

[50] Gholipour A., Rahmani S. (2023). The synthesis of fluorescent carbon quantum dots for tartrazine detection in food: A novel one-step microwave heating approach. Fuller. Nanotub. Carbon Nanostruct..

[51] Zhuang Q., Li L., Ding Y., Zeng H., Wu Y. (2019). Highly luminescent nitrogen-doped carbon dots as “turn-on” fluorescence probe for selective detection of melamine. ChemistrySelect.

[52] Wei Q., Liu T., Pu H., Sun D.W. (2020). Determination of acrylamide in food products based on the fluorescence enhancement induced by distance increase between functionalized carbon quantum dots. Talanta.

[53] Feng X., Han G., Cai J., Wang X. (2022). Au@carbon quantum dots-MXene nanocomposite as an electrochemical sensor for sensitive detection of nitrite. J. Colloid Interface Sci..

[54] Wei Q., Zhang P., Liu T., Pu H., Sun D.W. (2021). A fluorescence biosensor based on single-stranded DNA and carbon quantum dots for acrylamide detection. Food Chem..

[55] Salman B.I. (2024). An innovative selective fluorescence sensor for quantification of hazardous food colorant Allura Red in beverages using nitrogen-doped carbon quantum dots. J. Fluoresc..

[56] Chaudhari S.S., Patil P.O., Mali S.S., Alam M.S., Nangare S.N., Bari S.B., Khan Z.G. (2025). Design and synthesis of surface-decorated zinc-doped carbon quantum dots as fluorescent probes for tartrazine detection in real food samples exploiting the inner filter effect mechanism. Food Control.

[57] Huang Z., Wu C.T., Li J., Li L.S. (2026). One-step synthesis of dual-emission carbon dots for ratiometric fluorescence sensing of curcumin in food samples. Microchem. J..

[58] Liu L., Mi Z., Huo X., Yuan L., Bao Y., Liu Z., Feng F. (2022). A label-free fluorescence nanosensor based on nitrogen and phosphorus co-doped carbon quantum dots for ultra-sensitive detection of New Coccine in food samples. Food Chem..

[59] Liu J., Jiang H., Zhao C., Li H., Li Z., Zhang R. (2026). One-step microwave-assisted synthesis of nitrogen-doped carbon quantum dots for highly sensitive and selective fluorescence detection of mercury (II) ion in food. Food Chem. X.

[60] Mei H., Zhu X., Li Z., Jiang J., Wang H., Wang X., Zhou P. (2024). Manganese dioxide nanosheet-modulated ratiometric fluoroprobe based on carbon quantum dots from okra for selective and sensitive dichlorvos detection in foods. Food Chem..

[61] Adeyemi Oladipo A. (2024). Cu^0^-doped graphitic carbon quantum dots for rapid electrochemical sensing of glyphosate herbicide in environmental samples. Microchem. J..

[62] Pu Q., Zhang L., Ye N., Xiang Y. (2024). Lanthanide coordination carbon quantum dots construct a ratiometric fluorescent sensor for the detection of norfloxacin in milk. Dyes Pigments.

[63] Lu X., Gao Q., Xu L., Ren Y., Tong F., Wang Y., Wang L., Ge N., Ge W. (2025). Rapid determination of tetracycline utilizing a ratiometric fluorescence with Co-doped carbon quantum dots. J. Food Compos. Anal..

[64] Hu X., Shi J., Shi Y., Zou X., Arslan M., Zhang W., Huang X., Li Z., Xu Y. (2019). Use of a smartphone for visual detection of melamine in milk based on Au@carbon quantum dots nanocomposites. Food Chem..

[65] Peng B., Wang C., He X., Ma Y., Zhou M., Ma X., Zhao S., Fang Y. (2023). A smartphone-assisted ratiometric colorimetric and fluorescent probe for triple-mode determination of nitrite based on MnO_2_ nanoparticles and carbon quantum dots. Food Chem..

[66] Zhang X., Ren D., Wei Y., Ma H. (2025). Red light, green chemistry: On-site nitrite detection in pre-processed vegetables using diazotization-active carbon dots and smartphone-enabled dual-mode fluorometric/colorimetric readout. Anal. Chim. Acta.

[67] Kumari A., Sajwan R.K., Lakshmi G.B.V.S., Solanki P.R. (2024). A selective detection of tetracycline in animal-derived food samples using carbon quantum dots as a fluorescent eye. ACS Sustain. Resour. Manag..

[68] Kamyabi M.A., Moharramnezhad M. (2022). A novel cathodic electrochemiluminescent sensor based on CuS/carbon quantum dots/g-C_3_N_4_ nanosheets and boron nitride quantum dots for the sensitive detection of organophosphate pesticide. Microchem. J..

[69] He L., Du H. (2023). Detection of tartrazine with fluorescence sensor from crayfish shell carbon quantum dots. J. Food Compos. Anal..

[70] Leticia Almada-Leyva M., Tecuapa-Flores E.D., Garcia Rojas L.M., Thangarasu P. (2024). Ionic liquid and ZnO/carbon quantum dots derived from cat hair as an electrochemical sensor for ciprofloxacin in food samples: Experimental and cell-imaging studies. Electroanalysis.

[71] Yang Y., Xu J., Zhou R., Qin Z., Liao C., Shi S., Chen Y., Guo Y., Zhang S. (2024). Coordinated carbon dots-Fe featuring synergistically enhanced peroxidase-like activity at neutral pH for specific and background-free detection of dichlorvos. Carbon.

[72] Zhang J., Cai J., Wang B. (2022). A label-free fluorescent sensor based on Si,N-co-doped carbon quantum dots with enhanced sensitivity for the determination of Cr(VI). Materials.

[73] Phan T.Q., Vu T.B., Ngo H.D., Lam V.Q., Thuy V. (2025). Hydrothermal synthesis carbon dots derived from Blumea lacera for heavy metal detection. Mater. Res. Innov..

[74] Gencer Ö. (2025). The production of carbon quantum dots (CQDs) from cultivated blue crab shells and their application in the optical detection of heavy metals in aquaculture. Aquac. Int..

[75] Chaudhary N., Verma D., Yadav A.K., Sharma J.G., Solanki P.R. (2023). Rational hydrothermal-assisted green synthesis of blueish emitting carbon dots as an optical sensing platform for antibiotic detection in the milk sample. Talanta Open.

[76] Ameen S.S.M., Algethami F.K., Omer K.M. (2026). Doping-enhanced luminescence of biowaste-derived carbon quantum dots for dual-mode ratiometric and colorimetric detection of tetracycline. J. Inorg. Organomet. Polym. Mater..

[77] Wang X., Wang J., Hu B., Zhang Y., Tian H. (2024). Rapid determination of organophosphorus insecticides residue in fruit juice by a fluorescence sensor based on carbon quantum dots. J. Food Compos. Anal..

[78] Zhang R., Zhang L., Yu R., Wang C. (2023). Rapid and sensitive detection of methyl parathion in rice based on carbon quantum dots nano-fluorescence probe and inner filter effect. Food Chem..

[79] Zhang L., Chen J., Zhang L., Yu R. (2025). Rapid detection of chlorpyrifos in miscellaneous beans based on nitrogen and phosphorus doped carbon quantum dots fluorescence probe. J. Food Compos. Anal..

[80] Sinha N., Ray S. (2024). Application of carbon quantum dots derived from waste tea for the detection of pesticides in tea: A novel biosensor approach. ACS Omega.

[81] Wei L., Zhou B., Cai M., Wang X., Zhang G., Cheng X. (2025). Amide carbon quantum dots as chemiluminescent probes for the real-time quantitative detection of the carcinogen ethylene thiourea and its homologs in vegetables. Food Chem..

[82] Mehmandoust M., Erk N., Karaman O., Karimi F., Bijad M., Karaman C. (2021). Three-dimensional porous reduced graphene oxide decorated with carbon quantum dots and platinum nanoparticles for highly selective determination of azo dye compound tartrazine. Food Chem. Toxicol..

[83] Maurya S.K., Poonia A., Tiwari A. (2026). Green synthesis of carbon quantum dots from Sapindus mukorossi seeds and their application in fluorescence-based detection of chlorpyrifos in milk. Food Humanit..

[84] Jiang Y., Liu G., Jia H., Zhang Y., Tian H. (2026). A fluorescent sensor for rapid detection of three insecticide residues using N, S-doped carbon quantum dots. ChemistrySelect.

[85] Liu H., Yang J., Sun X., Wu P., Wang G., Huang Y., Li L., Ding Y. (2024). An advanced molecularly imprinted photochemical sensor based carbon quantum dots for highly sensitive detection of chloramphenicol in food. J. Fluoresc..

[86] Vadia F.Y., Ghosh S., Mehta V.N., Jha S., Malek N.I., Park T.J., Kailasa S.K. (2023). Fluorescence “turn OFF-ON” detection of Fe^3+^ and propiconazole pesticide using blue emissive carbon dots from lemon peel. Food Chem..

[87] Ashrafi Tafreshi F., Fatahi Z., Ghasemi S.F., Taherian A., Esfandiari N. (2020). Ultrasensitive fluorescent detection of pesticides in real sample by using green carbon dots. PLoS ONE.

[88] Song Y., Xu X., Yang L., Xu H. (2026). Carbon dot nanozymes driven ratiometric colorimetric and smartphone-assisted hydrogel for detection of nitrite in foods. Microchim. Acta.

[89] Shanker U., Anoushka, Rani M., Kaur N. (2025). Fluorescent detection of melamine in real samples using green-synthesized N-CQDs: A sustainable approach. Food Chem..

[90] Sajwan R.K., Kumar Himanshu J., Solanki P.R. (2024). Polyvinyl alcohol-derived-carbon quantum dots based fluorometric “on-off” probe for moxifloxacin detection in milk and egg samples. Food Chem..

[91] Das R.K., Singh G.P., Yadav D., Palwe A., Kumar C., Saxena S., Shukla S. (2025). Carbon quantum dots based nanocomposite for selective mercury detection. Small.

[92] Sajwan R.K., Solanki P.R. (2026). Carbon quantum dots/gold nanoparticle dual-mode aptasensor for smartphone-based gentamicin detection in food samples. ACS Appl. Nano Mater..

[93] Yue J.H., Zhou J.F., Zhu F.M., Li R.D., Liu X.J. (2025). Grape pomace carbon quantum dot-based dual-channel fluorescence sensor for sensitive detection of antibiotic residues in food. Food Anal. Methods.

[94] Ashkar M.A., Joseph R., Kutti Rani S., Vasimalai N. (2024). A novel molecular imprinted polymer grafted on N, S Co-doped carbon quantum dots-based fluorescence sensor for chloramphenicol in food and clinical samples. Microchem. J..

[95] Zhao J., Song Q., Wu F., Guo X., Xu T. (2019). Green synthesis of N-doped carbon quantum dots for the detection of nitrite ion in water sample. IOP Conf. Ser. Earth Environ. Sci..

[96] Cao Y., Wang X., Bai H., Jia P., Zhao Y., Liu Y., Wang L., Zhuang Y., Yue T. (2022). Fluorescent detection of tetracycline in foods based on carbon dots derived from natural red beet pigment. LWT.

[97] Mansouri S., Boulares S., Chabchoub S., Alharbi Y., Alqahtani A. (2025). Recent progress of smartphone-assisted paper-based analytical devices (PADs) for multiplex sensing: Focusing on colorimetric and optical sensors for environmental monitoring, food safety, and biomedical application. Microchem. J..

[98] Rizvi A.H., Fatima R., Ahmad A., Aziz M.A. (2025). Enhanced and rapid detection of mercury ions using one-step microwave synthesized fluorescent carbon quantum dots: Development of a very simple paper-based sensor with detailed mechanistic insights. Microchem. J..

[99] Kundu A., Basu S., Maity B. (2023). Upcycling waste: Citrus limon peel-derived carbon quantum dots for sensitive detection of tetracycline in the nanomolar range. ACS Omega.

[100] Vyas T., Joshi A. (2024). Carbon quantum dots (CQDs)-diphenyl carbazone (DPCO) loaded thin film sensors for fluorescent and colorimetric dual mode detection of mercury in various water resources. Opt. Mater..

[101] Vyas T., Joshi A. (2024). Chemical sensor thin film-based carbon quantum dots (CQDs) for the detection of heavy metal count in various water matrices. Analyst.

[102] Amer W.A., Rehab A.F., Abdelghafar M.E., Torad N.L., Atlam A.S., Ayad M.M. (2021). Green synthesis of carbon quantum dots from purslane leaves for the detection of formaldehyde using quartz crystal microbalance. Carbon.

[103] Ayad M.M., Abdelghafar M.E., Torad N.L., Yamauchi Y., Amer W.A. (2023). Green synthesis of carbon quantum dots toward highly sensitive detection of formaldehyde vapors using QCM sensor. Chemosphere.

[104] Liang N., Hu X., Li W., Mwakosya A.W., Guo Z., Xu Y., Huang X., Li Z., Zhang X., Zou X. (2021). Fluorescence and colorimetric dual-mode sensor for visual detection of malathion in cabbage based on carbon quantum dots and gold nanoparticles. Food Chem..

[105] Singh A., Singh G., Kaur N., Singh N. (2023). Fabrication of FRET based nano sensor from biomass-derived fluorescent carbon quantum dots and naphthalimide for ratiometric detection of nitric oxide: To examine nitrite levels in meat samples. Anal. Chim. Acta.

[106] Zhu X., Qi W., Wang M., Zhan S., Liu X., Zhao Y., Hessel V., Chen Z., Lin L. (2023). Microfluidic steam-based synthesis of luminescent carbon quantum dots as sensing probes for nitrite detection. Green Process. Synth..

[107] Murugesan P., Moses J.A. (2024). Carbon quantum dots stabilized silver nanoparticles-based colorimetric sensor for detection of pesticide residues. Mater. Sci. Eng. B.

[108] Pawar R.V., Patil P.O., Khalid M., Wahab S., Taleuzzaman M., Pardeshi S.R., Khan Z.G. (2025). Design of a facile fluorescent nano-sensor using nitrogen and sulfur dual doped carbon quantum dots for carbendazim detection: A turn-off-on approach for food safety and environmental monitoring. J. Fluoresc..

[109] Chen S., Ouyang W., Zhu Y., He L., Zou L., Ao X., Liu S., Yang Y., Li J. (2022). Facile synthesis of N, S-doped carbon quantum dots from food waste as fluorescent probe for sensitive detection of thiamphenicol and its analogues in real food samples along with an application in bioimaging. Foods.

[110] Li R., Yue J., Zhu F., Zhou J., Liu X. (2025). Application progress of carbon quantum dot composites in fluorescent detection of food safety. J. Food Sci..

[111] Sharma N., Thakur S., Bains A., Goksen G., Ali N., Ansari M.A., Kopsacheili A., Proestos C., Chawla P. (2025). Green hydrothermal approach for the synthesis of carbon quantum dots from waste tea bags for acrylamide detection in drinking water: A fluorescence assay validated by HPLC-PDA analysis. Food Chem. X.

[112] Chu H., Yao D., Chen J., Yu M., Su L. (2021). Detection of Hg^2+^ by a dual-fluorescence ratio probe constructed with rare-earth-element-doped cadmium telluride quantum dots and fluorescent carbon dots. ACS Omega.

[113] Liao S., Zhang L., Li S., Yue S., Wang G. (2023). Selective detection of mercury ions via single and dual signals by silicon-doped carbon quantum dots. New J. Chem..

[114] Mohammed Ameen S.S., Bedair A., Hamed M., Mansour F.R., Omer K.M. (2024). Repurposing expired metformin to fluorescent carbon quantum dots for ratiometric and color tonality visual detection of tetracycline with greenness evaluation. Microchem. J..

[115] Hama Aziz K.H., Omer K.M., Hamarawf R.F. (2019). Lowering the detection limit towards nanomolar mercury ion detection via surface modification of N-doped carbon quantum dots. New J. Chem..

[116] Ye Z., Zhang Y., Li G., Li B. (2020). Fluorescent determination of mercury(II) by green carbon quantum dots synthesized from eggshell membrane. Anal. Lett..

[117] Wang S., Wang W., Li M., Zhang L., Cai C., Niu B. (2022). On-site, rapid, and facile determination of gentamicin using a fluorescent resonance energy transfer sensor constructed from nitrogen-carbon quantum dots functionalized by 4,5-imidazole dicarboxylic acid. Food Chem..

[118] Li Q., Fu Y. (2025). Brilliant cresyl blue modified carbon quantum dots for sequential detection of insecticides fipronil, Fe^3+^, and acephate. Microchem. J..

[119] Yin X., Yang S., Du G., Zhang L., Xiang Y., Ye N. (2025). Exploration of the fluorine-fluorine interaction mechanism in fluoroquinolone antibiotics recognition and ciprofloxacin detection on the basis of fluorine-doped carbon quantum dots and machine learning. Food Chem..

[120] Chen Z., Huang H., Chen Y., Ye Y., Wu S., Wang W., Hu Y. (2020). Electrochemical detection of bisphenol A based on N-doped carbon quantum dots@carbon nanotubes composite. J. Nanosci. Nanotechnol..

